# “I Use Weed for My ADHD”: A Qualitative Analysis of Online Forum Discussions on Cannabis Use and ADHD

**DOI:** 10.1371/journal.pone.0156614

**Published:** 2016-05-26

**Authors:** John T. Mitchell, Maggie M. Sweitzer, Angela M. Tunno, Scott H. Kollins, F. Joseph McClernon

**Affiliations:** 1 Department of Psychiatry & Behavioral Sciences, Duke University Medical Center, Durham, North Carolina, United States of America; 2 Duke Center for Addiction Science and Technology, Durham, North Carolina, United States of America; University Children's Hospital Tuebingen, GERMANY

## Abstract

**Background:**

Attention-deficit/hyperactivity disorder (ADHD) is a risk factor for problematic cannabis use. However, clinical and anecdotal evidence suggest an increasingly popular perception that cannabis is therapeutic for ADHD, including via online resources. Given that the Internet is increasingly utilized as a source of healthcare information and may influence perceptions, we conducted a qualitative analysis of online forum discussions, also referred to as threads, on the effects of cannabis on ADHD to systematically characterize the content patients and caregivers may encounter about ADHD and cannabis.

**Methods:**

A total of 268 separate forum threads were identified. Twenty percent (20%) were randomly selected, which yielded 55 separate forum threads (mean number of individual posts per forum thread = 17.53) scored by three raters (Cohen’s kappa = 0.74). A final sample of 401 posts in these forum threads received at least one endorsement on predetermined topics following qualitative coding procedures.

**Results:**

Twenty-five (25%) percent of individual posts indicated that cannabis is therapeutic for ADHD, as opposed to 8% that it is harmful, 5% that it is both therapeutic and harmful, and 2% that it has no effect on ADHD. This pattern was generally consistent when the year of each post was considered. The greater endorsement of therapeutic versus harmful effects of cannabis did not generalize to mood, other (non-ADHD) psychiatric conditions, or overall domains of daily life. Additional themes emerged (e.g., cannabis being considered sanctioned by healthcare providers).

**Conclusions:**

Despite that there are no clinical recommendations or systematic research supporting the beneficial effects of cannabis use for ADHD, online discussions indicate that cannabis is considered therapeutic for ADHD—this is the first study to identify such a trend. This type of online information could shape ADHD patient and caregiver perceptions, and influence cannabis use and clinical care.

## Introduction

Cannabis use disorder (CUD) refers to a problematic pattern of cannabis use leading to clinically significant impairment or distress within a 12 month period and includes at least two symptoms occurring in this context (e.g., cannabis being taken in a larger amount or over a longer period than was intended, unsuccessful attempts to control use, a strong desire to use cannabis, and recurrent use resulting in failure to fulfill major life obligations) [1]. Individuals with attention-deficit/hyperactivity disorder (ADHD) are at increased risk for both cannabis use and CUD compared to the general population. In the largest meta-analysis to date examining the prospective association of ADHD with cannabis use, ADHD youth were nearly three times as likely to report cannabis use in later life compared to non-ADHD youth; and ADHD children were more than 1.5 times as likely to be subsequently diagnosed with a CUD [2]. In a large, multisite longitudinal study, individuals initially diagnosed with ADHD between the ages of 7–9 years were significantly more likely than controls to report cannabis use at 8-year follow-up (32.1% and 24.0% for ADHD and non-ADHD, respectively) [3]. ADHD adolescents were more likely to meet criteria for a CUD as well, which persisted into early adulthood [4]. Conversely, in samples with a CUD, comorbidity with ADHD ranges from 33%-38% [5]. Even in non-clinical samples, ADHD symptoms are associated with increased cannabis use severity, craving, abuse, dependence, and earlier initiation of use [6, 7].

This relationship between ADHD and cannabis use is relevant given the known adverse effects of use. For instance, short-term effects of cannabis use include impaired short-term memory and motor coordination, altered judgement, and (in high doses) paranoia and psychosis [8]. Real-world outcomes of such effects include higher rates of motor vehicle accidents. The effect of long-term or heavy use include altered brain development, poorer educational outcomes (e.g., higher likelihood of dropping out of school), lower intelligence, diminished life satisfaction, symptoms of chronic bronchitis, and increased risk for chronic psychosis disorders in people with a predisposition to such disorders [8]. Cardiovascular disease, poorer mental health, use of other illicit substances, and a range of poorer neurocognitive outcomes (e.g., attention, executive functioning, and inhibition) have also been identified [9–14]. Given that similar outcomes are associated with ADHD *independent of cannabis use*, including neurocognitive deficits [15, 16] and poor driving [17], the maladaptive effects of cannabis use may be particularly pronounced in ADHD patients. Indeed, heavier cannabis use in people with ADHD appears to have an additive effect on poor neurocognitive outcomes [18] and alters hippocampal and cerebellar-dependent function [19], along with frontal and postcentral cortical thickness [20]. Further, this comorbidity will likely impact successful treatment of cannabis use since ADHD symptoms are correlated with cannabis craving [7] and such craving is associated with relapse [21].

Despite the increased risk for problematic cannabis use outcomes, anecdotal clinical observations suggest there is a growing popular perception that cannabis is therapeutic for ADHD. Even some medical professionals have advocated for cannabis as a treatment for ADHD, including before a congressional subcommittee on drug policy [22] (also see [23]). Consistent with such observations, the perceived risk associated with regular cannabis use in the general population has decreased among adolescents and young adults to its lowest point since the late 1970s [24], which is paralleled by patients and caregivers increasingly inquiring about the therapeutic effects of cannabis for developmental and behavioral disorders [25]. It is particularly relevant to address factors that may influence perceptions about the effects of cannabis on ADHD given that substance use perceptions can influence use [26, 27] and, as noted above, cannabis has adverse effects, especially for at-risk populations such as those with ADHD. As legalized recreational use among adults could significantly increase access to cannabis among youth and is a growing concern for pediatric health in the US [28], identifying factors that may impact perceptions promoting cannabis use is a timely issue that is likely to become increasingly important.

The overall aim of this study was to systematically characterize one source of information that patients and caregivers may use to inform their opinions about ADHD and cannabis: the Internet. Approximately 72% of adult [29] and 84% of adolescent [30] US Internet users query the Internet for healthcare information. Analysis of online information has been increasingly used to identify emerging patterns of substance use [31–34], though this has not been extended to substance use in ADHD. Online forums in particular were selected for this study since psychiatric and substance use populations report using forums to inform their healthcare decisions [35, 36]. Indeed, more individuals indicate they are more likely to use online forums to address mental health concerns than face-to-face with another person [37]. These forums facilitate social interactions and allow individuals to self-disclose their unfiltered experiences, inquiries, and opinions about substance use in an anonymous format [34, 38–43], and may be a fruitful starting point for understanding what patients and caregivers are exposed to when searching for information about the effects of cannabis on ADHD.

A qualitative methodology was adopted for the current study to examine the content of online forum threads on the topic of ADHD and cannabis use to identify trends in comments about their relation, particularly regarding therapeutic and adverse effects of cannabis on ADHD. This is an important topic since ADHD patients and caregivers may use such online resources to learn about and inform treatment decisions for ADHD. There are no studies examining how ADHD and cannabis are portrayed online, therefore we adopted a largely exploratory approach to identify trends in forum content that will inform future studies. However, based on changes in the perceived risks of cannabis use [24], patients and caregivers increasingly inquiring about the therapeutic effects of cannabis [25], and anecdotal clinical observations on the relationship between ADHD and cannabis, we hypothesized that the majority of forum posts would advocate for the therapeutic effects of cannabis for ADHD in comparison to harmful effects.

## Methods

### Sample and Procedure

A qualitative descriptive methodological approach was adopted. As outlined in Flower et al. [44], this approach can be utilized to examine naturalistic language to characterize perceptions and experiences with a particular topic that is poorly understood [45, 46]; however, the aim of this study was to examine online forum content patients and caregivers may be exposed to as opposed to characterize perceptions of forum members. To identify forum threads, past studies [44, 47] have collected posts from a particular online forum dedicated to a specific patient population. We attempted to expand upon this approach and sample a variety of online forums that patients with ADHD and caregivers may come across when conducting a search for discussions on the topic of ADHD and cannabis.

A search via Google, the most frequently used online search engine in the US [48], was conducted on 10/28/14-10/29/14 using every combination of three different groups of search terms: (a) “ADHD,” “ADD,” or “attention deficit” *with* (b) “marijuana,” cannabis,” “pot,” or “weed,” *with* (c) “forum.” At least the first 50 results that emerged from each search were considered. Forum threads that included links to any other forum threads addressing ADHD and cannabis were also included. This resulted in a total sample of 268 forum threads identified for the current study.

We randomly selected 55 threads (20%) for analysis, which were coded for the presence or absence of particular topics (see Qualitative Coding below). The average number of individual posts within each forum thread was 17.63 (*SD* = 17.22, range = 1 to 85, median = 13, mode = 4). A random selection of 20% is consistent with other qualitative studies of online forums on the topic of substance use [47] and yields a relatively higher number of individual posts than other studies on online forums [49, 50]. The 55 threads yielded a total of 964 individual posts. Among these 55 threads, 9 (16%) did not contain any individual posts that received at least one endorsement for the topics coded for this study. Such threads included wording used in our search, but did not actually include any comments on any aspect of the relationship between ADHD and cannabis use. Removal of these 9 threads (totaling 84 individual posts) resulted in 46 threads for analysis. These 46 threads contained 880 individual posts. The average number of individuals posts within each forum thread was 19.13 (*SD* = 17.97, range = 1 to 85 posts, median = 15, mode = 15). Among the 46 threads, 27 (59%) were hosted on sites devoted to ADHD generally (e.g., http://www.addforums.com, http://www.adhdmarriage.com), 10 (22%) were hosted on sites devoted to cannabis generally (http://marijuana.com, http://www.rollitup.org), 6 (13%) were hosted on sites devoted to overall physical or mental health generally (http://ehealthforum.com, http://www.psychforums.com), and 3 (7%) were hosted on other sites (http://www.thetechgame.com, http://www.econjobrumors.com).

**Fig 1:**
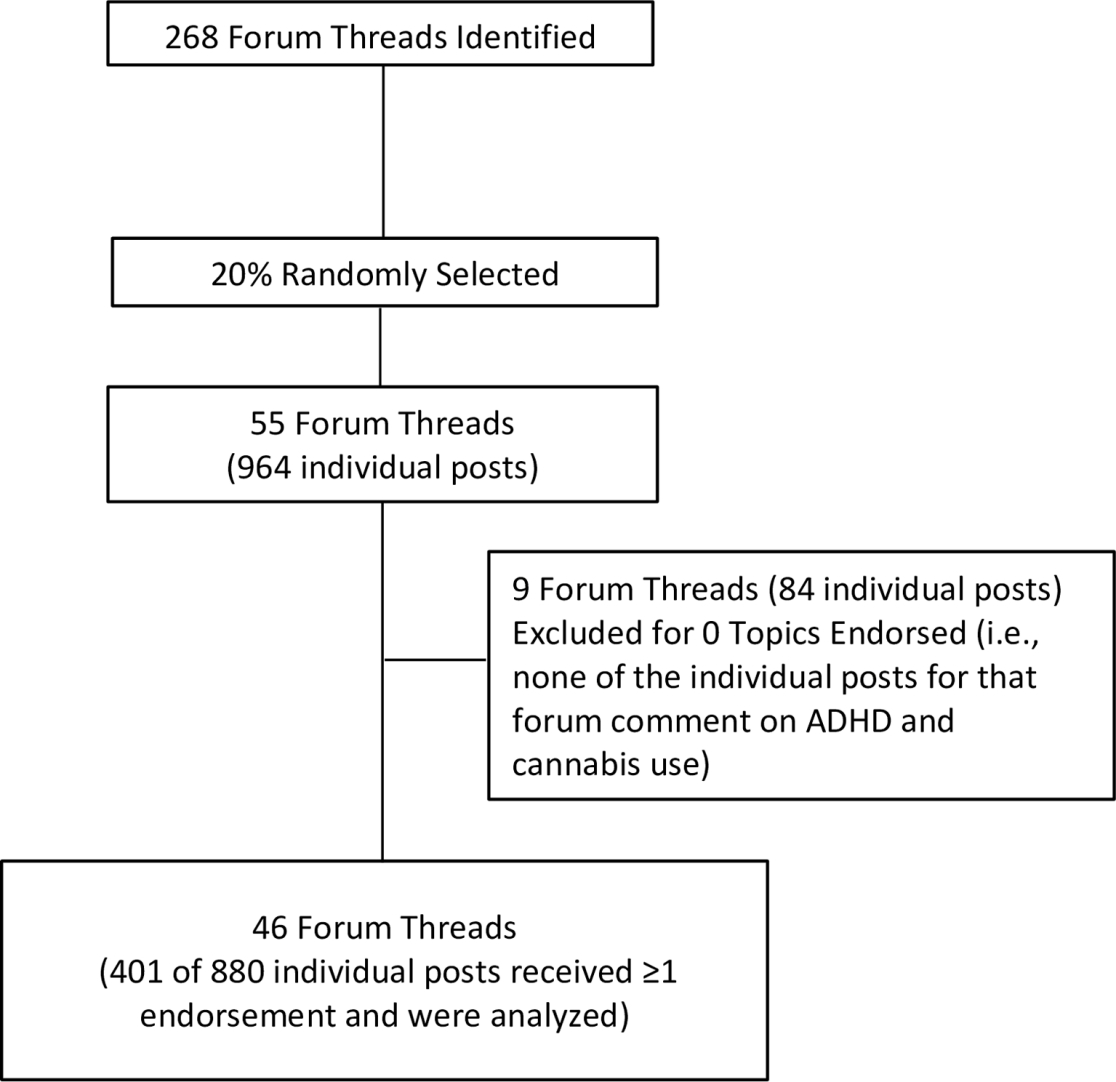
Internet Forum Identification Summary.

A total 479 of the 880 individual posts within the 46 threads did not receive an endorsement for various reasons, such as thanking others for commenting on a question, introducing themselves to the discussion, or tangentially commenting on a discussion about cannabis and ADHD (e.g., stating that they don’t use cannabis, but smoke cigarettes). These posts were excluded given the primary aim of this study to characterize the content of comments on ADHD and cannabis. A total of 401 individual forum posts received at least one topic endorsement within the 46 threads (see Qualitative Coding below). See Fig 1 for a summary. To allow for analysis of temporal distribution of forum threads, the year of each post was recorded. This study was exempted from human subjects review by the Duke Institutional Review Board due to the anonymous and public-access format of the source data.

### Qualitative Coding

A list of topics for coding individual posts in a binary response format (i.e., each post received either a “1” for an endorsement or “0” for a non-endorsement for topics such as “Cannabis helps with attention, hyperactivity-impulsivity, or ADHD”) was created through an iterative process. First, a list of potential codes was created *a priori* by the authors for anticipated online forum discussions involving cannabis and ADHD based on knowledge of the literature and aims of this study. This was followed by a review of 266 individual posts across 30 different threads, prior to random selection of posts, to assess topics that emerged that were not previously considered in the first stage. The authors then finalized the list of specific topics that were coded based on the primary aims of the current study—additional topics on the subject of ADHD and cannabis were coded as well, but are not reported in this study. All 401 individual posts analyzed in this study received at least one endorsement from any of the topics on the subject of ADHD and cannabis used in the coding process (including the additional topics not reported on in this study).

Among the topics that were coded for the current study, we assessed if cannabis was stated to impact ADHD or ADHD symptoms, mood, non-ADHD psychiatric disorders, and different domains of daily living (i.e., sleep, driving, social functioning, motivation, academic performance, and general quality of life). For each of these topics, coding was carried out to indicate if the effect of cannabis was stated to be therapeutic, harmful, or both therapeutic and harmful. For the code on the effect of cannabis on ADHD, a null effects option (i.e., the post specifically stated there is no effect of cannabis on ADHD) was also included—null effects were not coded for cannabis effects on mood, non-ADHD psychiatric disorders, or different domains of daily living given that this endorsement option was not observed in the code creation process. Comments about medicinal aspects of cannabis in the context of ADHD were also coded (see Table 1).

**Table 1:** Topic Endorsement Summary Among Forum Thread Posts.

					% Topic Endorsed
Impact of cannabis on ADHD or ADHD symptoms					
	Therapeutic				25%
	Harmful				8%
	Therapeutic and harmful				5%
	Null effect				2%
Impact of cannabis in other domains					
	Mood				
		Therapeutic			14%
		Harmful			13%
		Therapeutic and harmful			3%
	Other psychiatric conditions				
		Therapeutic			10%
		Harmful			8%
		Therapeutic and harmful			1%
	Different domains of daily life (e.g., sleep)				
		Therapeutic			11%
		Harmful			7%
		Therapeutic and harmful			4%
Comments about cannabis as medicinal					
	Cannabis more effective than ADHD medications				5%
	Cannabis less effective than ADHD medications				3%
	Reference to cannabis as medicinal or sanctioned by healthcare providers				15%

*Notes*. Percentage calculations based on a denominator of 401.

The randomly selected 20% of forum threads were all read in full by one of three raters. From these forum threads, 401 individual posts received at least one code endorsement by a rater. In cases where a person posting quoted another post that was endorsed but they themselves did not clearly endorse or provide sufficient information for the rater to determine if an endorsement was warranted for a particular code, then an endorsement was not made by the rater. Multiple posts by the same person (as indicated by a user identification name or number) were allowed since the main purpose of this study was to assess what forum users might be exposed to when looking for information on the topic of ADHD and cannabis use, therefore multiple posts by the same person would not have a meaningful impact on interpretation of the results.

A random selection of 10% of posts was coded by the other two raters for inter-rater reliability. The average agreement between each grouping of raters was 93% (range: 92%-93%). Cohen’s kappa takes chance agreement into account and was 0.74 (ranging from 0.72 and 0.76 between different pairs of raters), indicating substantial agreement [51].

Examples that typified endorsements for different topics are reported. Spelling and grammatical errors were not corrected, although vague use of pronouns (e.g., “it”) were replaced with specific terms used elsewhere in the post (e.g., “medical marijuana”) and denoted by use of parentheses. Also, use of “…” within quotes indicates sections that were removed to allow for brevity while maintaining the overall context of the quote.

## Results

Table 1 shows that out of the 401 individual posts examined, 25% (99 posts) endorsed that cannabis improved ADHD or ADHD symptoms, compared to 8% that it is harmful (31 posts), 5% that it is both therapeutic and harmful (19 posts), and 2% that it has null effects (7 posts). We also compared posts proposing that cannabis is therapeutic to domains other than ADHD. Table 1 demonstrates that the higher percentage of posts supporting therapeutic versus harmful effects of cannabis was not as apparent for other outcomes (i.e., mood, other psychiatric conditions, general quality of life), which ranged from 1% (5 posts) to 14% (56 posts) of the 401 posts.

The majority of forums (59%) included posts that advocated for the therapeutic effects of cannabis for ADHD (i.e., 27 of 46 forum threads). In terms of comments about cannabis as therapeutic for ADHD or ADHD symptoms, below are examples that typified endorsements for this topic:

“Marijuana works for ADHD”

"(Cannabis) helps me greatly with my ADHD"

“In regard to the ADD, while you are high … you will be able to focus much, much better than you normally would.”

“medical marijuana improves the ability to concentrate in some types of ADD.”

“There are many, many studies showing the efficacy of (medical marijuana) for ADD.”

Regarding the relatively fewer posts about cannabis’ adverse effect on ADHD or ADHD symptoms, typical comments included:

“For me, pot does nothing for my ADD. If anything, it makes it worse. I cant pay attention sober much less high.”

“Smoking weed is bad for people with ADHD.”

Among the few that stated cannabis is both therapeutic and worsens ADHD, comments such as “*Cannabis both helps and makes (my ADHD symptoms) more intense*” emerged.

Within the 99 posts that endorsed that cannabis improved ADHD or ADHD symptoms, there were 62 posts that mentioned improvement on at least one DSM-5 ADHD symptom set (i.e., inattention or hyperactivity-impulsivity), as opposed to posts that stated that cannabis helped ADHD but not at the symptom level. Among the 62 posts that commented on ADHD symptoms, 74% (46 posts) endorsed that cannabis helped inattentive symptoms (e.g., “*(Cannabis) helps me focus*” and “*i find im able to concentrate so much better after a bit of cannabis*"), 16% (10 posts) that cannabis helped hyperactive-impulsive symptoms (e.g., *“(cannabis) sort of helps eliminate excess pent up hyperactivity*” and “*i just smoke (cannabis) everyday after school and then around night and im good i dont get to hyper and i have total control of how i act*”), and 10% (6 posts) that cannabis helped both inattentive and hyperactive-impulsive symptoms (e.g., “*when (I) attend class stoned … (I am) more focused and less nervous and hyperactive*” and “*(I) use pot for (ADHD)*, *and it helps quell racing thoughts*, *hyperactivity*, *and rage*, *while considerably extending attention span*.”).

**Fig 2:**
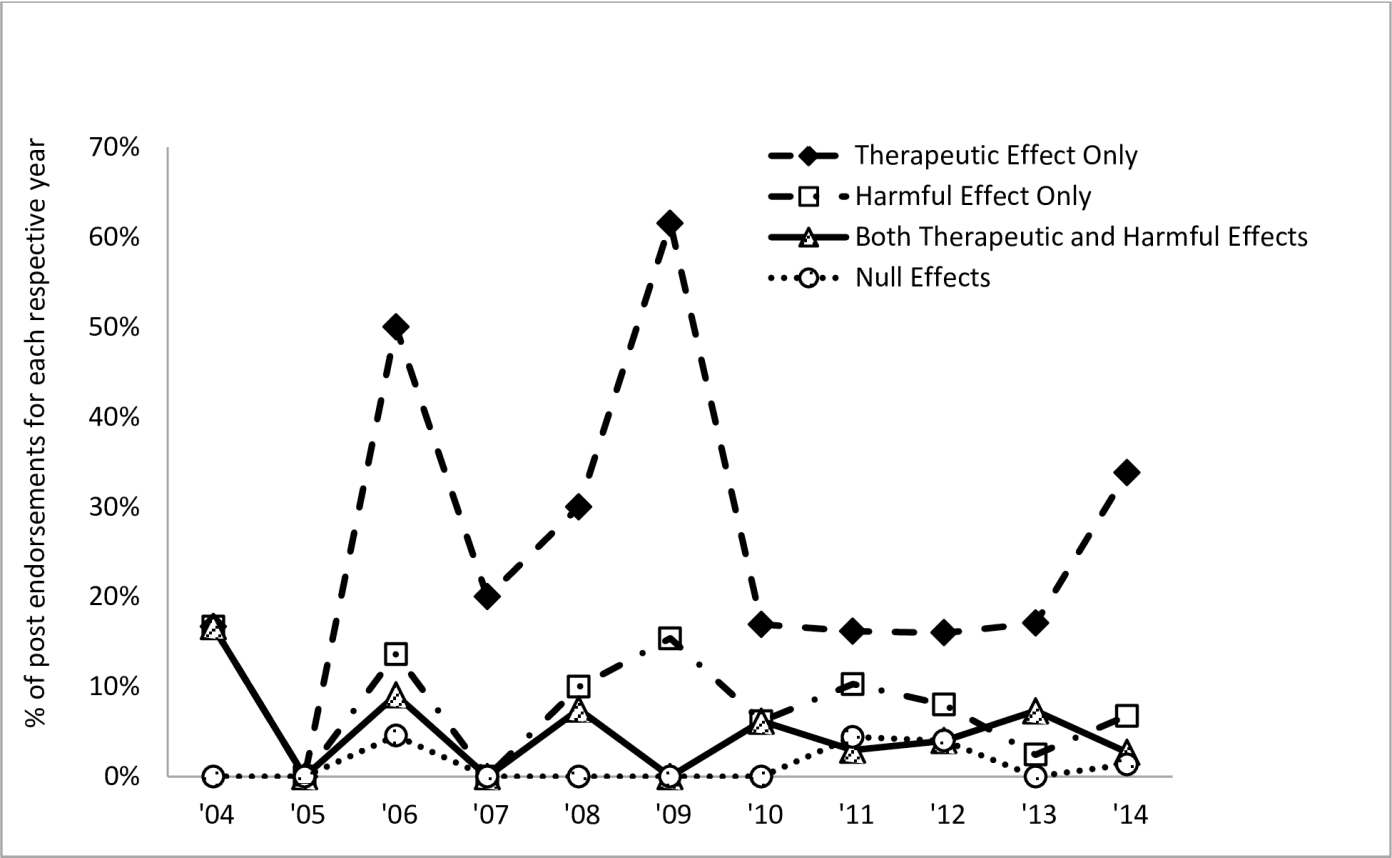
The percentage of individual post endorsements for each respective code on the effects of cannabis on ADHD per year (2004–2014) among the 401 posts analyzed.

We also evaluated changes in the content of posts involving impact of cannabis on ADHD or ADHD symptoms over time across the 401 posts. All 401 posts were made between 2004 and 2014. The percentage of individual post endorsements on the therapeutic, harmful, therapeutic and harmful, and null effects of cannabis on ADHD per year was considered (e.g., the number of endorsements that cannabis is effective for ADHD for a particular year divided by the total number of posts for that year). As shown in Fig 2, endorsements indicating that cannabis is therapeutic for ADHD has been consistently higher since 2006 relative to posts about its potential harmful impact, combined therapeutic and harmful effects, or null effect.

Comments about the medicinal aspects of cannabis use in the context of discussing ADHD were also considered across the 401 posts (Table 1). Overall, few commented on how cannabis compares to ADHD medications (i.e., 5% [22 posts] indicated that cannabis is more effective and 3% [12 posts] that it is less effective). Fifteen percent (62 posts) indicated that cannabis was considered medicinal or sanctioned by healthcare providers. In many cases, these posts pertained to the medicinal use of cannabis for ADHD. For example, one post stated “*I am 21 years of age and strongly believe in medical marijuana and why people with adhd should be getting percribed it in this country*” and then listed a link to a website from a physician purportedly advocating cannabis as a treatment for ADHD. Other examples of posts that typified this topic include:

“(A physician) has also worked with one family of a 15-year-old—whose family had tried every drug available to help their son, who by age 13 had become a problem student diagnosed as suffering from ADHD. Under (a physician’s) supervision, he began marijuana treatment, settling on cannabis in food and candy form, and he has since found equilibrium and regularly attends school.”

“I have ADD and I use (medical marijuana) for it. It works very well. I'm 64 and was diagnosed with adult ADD 4 years ago. I had it confirmed by two MD's and a psychiatrist.…I now use marijuana exclusively.”

## Discussion

This study is the first to systematically analyze a source of online information that patients and caregivers may use to inform their opinions about ADHD and cannabis. A qualitative analysis examining the content of online forum threads on the topic of ADHD and cannabis use indicated that at least three times as many comments advocated for therapeutic effects of cannabis on ADHD compared to comments that cannabis is harmful, both therapeutic and harmful, or has no effect on ADHD. The disproportionate number of comments favoring the therapeutic over harmful effects of cannabis was specific to ADHD and was not generalizable when mood, non-ADHD psychiatric conditions, or general quality of life were considered. Analysis of the temporal distribution of posts about the effects of cannabis on ADHD indicated that the tendency to advocate for its therapeutic effects has generally been consistent since 2006. Qualitative analysis also indicated that comments purporting the therapeutic effects of cannabis for ADHD predominantly referenced improvement in inattentive symptoms, as opposed to hyperactive-impulsive symptoms. Relatively few comments comparing cannabis against ADHD medications emerged. However, there were a number of comments indicating that cannabis is considered “medicinal” or sanctioned by healthcare providers.

The primary motivation in conducting this analysis was to systematically identify and analyze a source of information patients and caregivers might access to learn about the effects of cannabis use on ADHD. The majority of US Internet users query the Internet for healthcare information [29]. This necessitates addressing patient use of the Internet in clinical practice and how it affects the patient-provider relationship [52]. Our data suggest that patients seeking information regarding cannabis effects on ADHD will find a greater amount of information on Internet forums biased toward cannabis improving ADHD. This is relevant for healthcare providers so that they can anticipate perceptions informed by online resources and develop a communication style that is both inclusive of patient concerns based on such searches and contributes to quality health care [53, 54].

Our findings indicating bias towards cannabis being beneficial for ADHD is consistent with national trends about the decreased perceived risk associated with regular cannabis use [24] and is relevant to individuals diagnosed with ADHD. Such online information may impact perceptions promoting use, which can be problematic given the maladaptive effects of use [8, 12–14]. In particular, the adverse neurocognitive effects of cannabis use [9–11] may have an additive effect on neurocognitive deficits observed in ADHD *independent of cannabis use* [15, 16]—emerging findings support this additive effect in those with ADHD [18]. Consequently, the actual effects of cannabis use may be particularly maladaptive in ADHD patients, which stands in stark contrast to messages arguing for therapeutic effects identified in this study. Further, there are no systematically collected data to support that cannabis is therapeutic for ADHD. Findings from this study are particularly relevant as nearly half of all US states have enacted legislation legalizing medicinal cannabis, while four states plus the District of Columbia have passed laws legalizing recreational use that will likely translate into greater cannabis availability to adolescents (and more certainly young adults).

The current findings demonstrate that an online source that may be used by caregivers and patients with ADHD seeking more information about cannabis favors the beneficial effects of use on ADHD. Future studies are needed to assess if these attitudes are endorsed by ADHD patients. To our knowledge, only one study has examined perceptions of cannabis use in ADHD [55], though this study did not assess the perceived impact on ADHD. If this is supported, future studies are needed to (a) assess if such perceptions predict cannabis use in ADHD samples and (b) compare these perceptions against objective effects of cannabis in ADHD samples. Increased understanding of these relationships can inform treatment of ADHD patients and cannabis prevention efforts (e.g., education about the inaccuracy of perceptions about the effects of cannabis use on ADHD symptoms).

In terms of study limitations, although data was collected from a resource that patients and caregivers may use to learn about cannabis and ADHD (i.e., online forums), it is unclear how often ADHD patients and caregivers access this online resource or online resources in general to learn more about treatment options. However, past studies do demonstrate that individuals seek out healthcare information online [29] and that this extends to inquiries about therapeutic effects of cannabis for different psychiatric disorders [25]. Another limitation is that this study only focuses on one Internet resource: forums. Although this study was therefore restricted in scope, forums are commonly used to inform mental health care decisions [35–37] and were therefore targeted for this study. Also, given the anonymous format of forum threads, there are no demographic data or information about diagnostic status available for individuals who posted information. Further, individuals who post on forums may be a self-selected sample that may not represent views of the general population of ADHD patients. However, this is not a limitation of the current study given that our main aim was to assess what is advocated through online forums, regardless of who is posting on such forums. Relatedly, no inferences can be drawn about the prevalence of perceptions regarding the effects of cannabis on ADHD in patients with the disorder—that was beyond the scope of the present study (i.e., to assess the content of online data referring to cannabis and ADHD in forums). To address this concern, as mentioned above, future studies that examine perceptions among well-characterized ADHD samples are needed.

## Conclusions

In summary, there is a dearth of systematic studies analyzing a source of online information patients and caregivers might access to learn about the effects of cannabis on ADHD—this is the first study, to our knowledge, to do so. Our findings involving the trend of online forum threads advocating for the therapeutic effects of cannabis for ADHD are particularly important since patients and caregivers seek out information online. Moreover, this topic is likely to be increasingly broached in clinical settings. This study also demonstrates the utility of online data to examine trends in substance use and inform future studies. In particular, findings from this study indicate the need to assess perceptions involving cannabis use and ADHD in ADHD patients, in addition to examining the objective effects of cannabis use on ADHD symptoms and associated features.

## Supporting Information

S1 Dataset The additional file “S1_Dataset” contains data. In this Excel file, we provide one file: S1_Dataset.xlsx.
(XLSX)
